# Primary Aspergillosis of Bilateral Laryngoceles

**DOI:** 10.1155/2014/384271

**Published:** 2014-02-03

**Authors:** Zeyad Al-Ogaili, Gavin Chapeikin, David Palmer

**Affiliations:** ^1^Department of Radiology, Royal Perth Hospital, Perth, WA 6000, Australia; ^2^Department of Radiology, Sir Charles Gairdner Hospital, Nedlands, WA 6008, Australia; ^3^Western Diagnostic Pathology, Myaree, WA 6154, Australia

## Abstract

Laryngocele is an abnormal dilatation of the saccule of laryngeal ventricle, which is usually unilateral and filled with air or fluid. We present a case of bilateral laryngoceles colonized by *Aspergillus* species.

## 1. Introduction

Laryngocele is an uncommon condition characterized by abnormal dilatation and elongation of the saccule of laryngeal ventricle of Morgagni [1]. It may be classified as internal, external, or mixed. The usual presentation is of a swelling in the upper neck and diagnosis is confirmed radiologically, primarily by means of computed tomography. Connection between the air sac and the airway helps to establish the diagnosis. Surgery is indicated for symptomatic laryngoceles.

Laryngocele can be air-filled or fluid-filled. Bacterial infection can result in pus formation (laryngopyocele). Colonization with fungal infection such as *Aspergillus* species was not previously described. Aspergillosis, which can be defined as an infection or disease caused by fungi in the genus *Aspergillus*, constitutes a wide range of disease entities that form a continuum from allergic reactions to disseminated invasive disease in immunocompromised patients [2].

## 2. Case Report

A 77-year-old lady presented to ENT clinic with 4-month history of worsening dysphagia to solids and liquids and difficulty of talking. Past medical history includes smoking and asthma for which she was on inhaled corticosteroids.

On examination, there was no stridor but the patient was dysphonic. Her oral cavity, oropharynx, and neck examination were unremarkable.

Fibreoptic nasoendoscopy revealed large bilateral supraglottic masses centered on the aryepiglottic folds. The right tumour was significantly larger than the left and encroached on the laryngeal inlet. The left vocal cord was mobile but the right cord was fixed. There was significant luminal narrowing with only 30% of the airway remaining visible.

**Figure 1:**
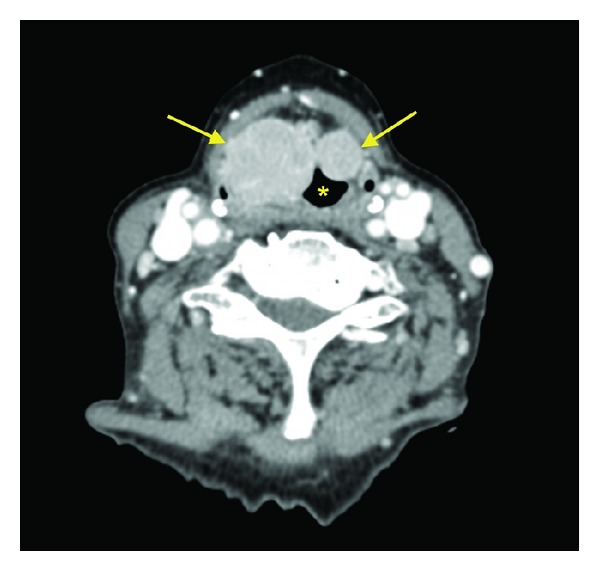
Axial contrast enhanced CT scan at the level of true vocal cords showing bilateral solid paraglottic masses (arrows) narrowing the laryngeal lumen (asterisk).

**Figure 2:**
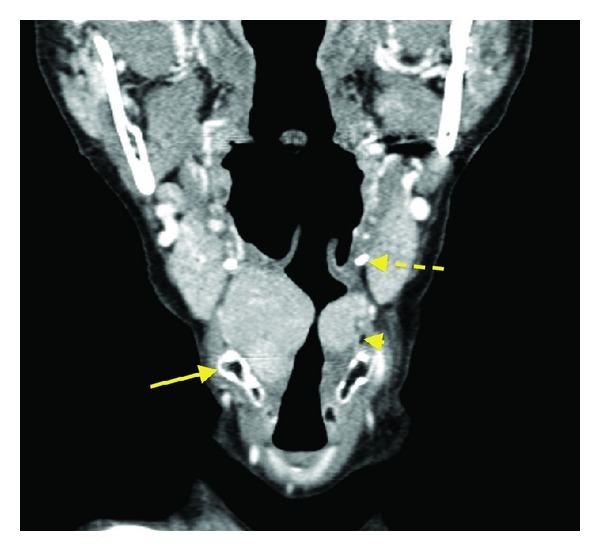
Both masses are medial to the thyrohyoid membrane in keeping with internal laryngoceles. Note how the pyriform sinus (arrow head) is almost completely effaced. Thyroid cartilage (arrow). Hyoid bone (interrupted arrow).

A CT scan of the neck (Figures 1 and 2) showed bilateral large well-defined homogenously hyperdense glottic and supraglottic masses, centered on the paralaryngeal fat and extending from the hyoid bone to the true cords.

A communication with the laryngeal ventricle was not clear; therefore, the initial impression was lymphoma or considered far less likely bulky laryngeal carcinoma.

**Figure 3:**
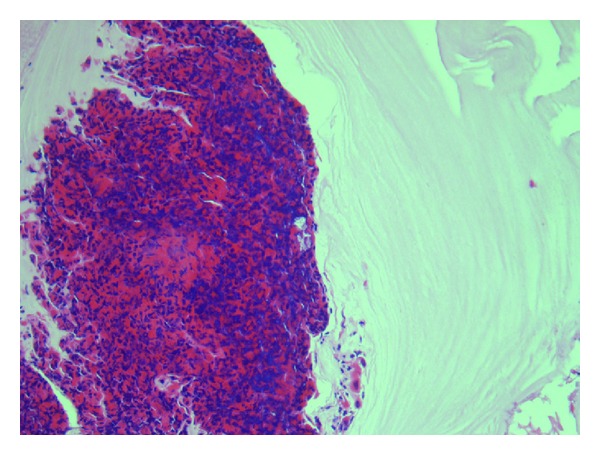
Aggregate of eosinophils with mucus. H & E ×200.

**Figure 4:**
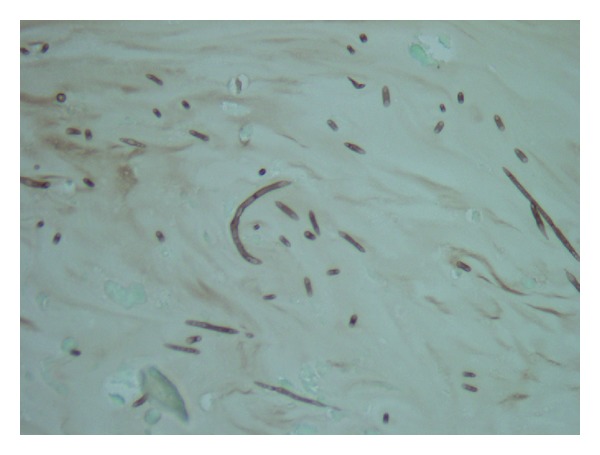
Collection of septate hyphae consistent with *Aspergillus* species. Methenamine silver, ×200.

The patient underwent a microlaryngoscopy and biopsy. The mass was incised. Surprisingly, fungal mucin was removed; the lesion was decompressed and sent for cytology, which confirmed fungal mucin (Figures 3 and 4). Culture revealed Aspergillus. Immediately postoperatively, the patient voice returned to normal and dysphagia resolved.

## 3. Discussion

Virchow first introduced the term laryngocele in 1867 to describe an abnormal dilatation of the appendix of the laryngeal ventricle of Morgagni, which maintains its communication with the laryngeal lumen [3]. Three types of laryngoceles have been described: internal, external, and mixed, the distinction is related to the position of the sac with respect to the thyrohyoid membrane. Laryngoceles are usually unilateral; however, bilateral laryngoceles occur in approximately one-fifth of cases [4]. Usually laryngoceles are either filled with air or fluid. If secondarily infected, they are then referred to as laryngopyoceles [5]. Primary Aspergillosis localized to the larynx is an extremely rare condition. The literature search over 30 years has produced fewer than 12 cases in total [6]. As far as we know, there is no report in the literature of Aspergillosis involving laryngocele.

The majority of laryngoceles are asymptomatic usually presenting in the fifth decade. When symptomatic, the presenting complaint is usually dysphonia, dyspnoea, or sore throat. Coexistence of laryngoceles and laryngeal carcinoma is still being debated and has been extensively reported in the literature.

Radiological diagnosis is primarily by means of CT scan, which can show the characteristic intralaryngeal and extralaryngeal expansion, the relationship with the laryngeal ventricle and thyroid membrane, and the presence of carcinoma. In case of laryngopyocele, a contrast-enhanced CT scan can demonstrate signs of inflammation such as thickening of the walls or enhancement of the laryngocele and assist the differential diagnosis.

The differential diagnosis of laryngopyocele includes laryngeal cyst, fluid-filled laryngocele, branchial cleft cyst and less likely paraganglioma, schwannoma, and thyroglossal duct cysts.

We present a previously nondescribed rare entity, which mimicked not only the above pathologies, but also the more sinister entity of laryngeal lymphoma.
